# The Cost of Annual and More Frequent Than Annual Mass Drug Administration for Trachoma in Two Districts in Amhara, Ethiopia

**DOI:** 10.4269/ajtmh.25-0498

**Published:** 2026-01-20

**Authors:** Tim Jesudason, Sayara Ahmed, Hugo C. Turner, Eshetu Sata, Ayalew Shiferaw, Teferi Demmelash, Adisu Abebe, Alethia Sanon, E. Kelly Callahan, Kimberly A. Jensen, Scott D. Nash

**Affiliations:** ^1^ Partners in Global Health Ltd, Victoria, Malta;; ^2^ The Carter Center, Addis Ababa, Ethiopia;; ^3^ Amhara Regional Health Bureau, Bahir Dar, Ethiopia;; ^4^ The Carter Center, Atlanta, Georgia

## Abstract

Ethiopia accounts for 59% of the global trachoma burden. To eliminate trachoma as a public health problem by 2030, modified mass drug administration (MDA) strategies have been proposed, including more frequent than annual MDA. In the present study, the cost of the “child MDA” (CMDA) strategy, defined as an initial community-wide MDA treatment followed by another treatment targeting children aged 6 months to 9 years, was estimated in the Lasta and Wadilla districts, Amhara, Ethiopia. A micro-costing analysis was conducted from a payer perspective, documenting the total financial and economic cost, cost per person treated, and cost per treatment. The cost per person treated was calculated by dividing the total cost by the total number of people treated during the community-wide MDA distribution. The cost per treatment was calculated by dividing the total cost by the total number of treatments distributed overall. The total financial cost of implementing the CMDA strategy in Lasta and Wadilla was $106,427, corresponding to a financial cost per person treated of $0.41 and a financial cost per treatment of $0.32. In Lasta, 168,175 treatments were distributed at a financial cost of $61,978, corresponding to a cost per person of $0.48 and a cost per treatment of $0.37. In Wadilla, 169,248 treatments were distributed at a financial cost of $44,449, corresponding to a cost per person of $0.34 and a cost per treatment of $0.26. This information is useful to stakeholders considering the CMDA strategy in similar contexts and may contribute to future cost-effectiveness analyses of the strategy.

## INTRODUCTION

Trachoma, caused by the bacterium *Chlamydia trachomatis*, is the world’s leading infectious cause of blindness.^1^ Significant progress has been made to reduce the global burden of trachoma, including a 93% reduction in the number of people at risk, from 1.5 billion in 2002 to 103 million in 2024.^1^ Despite this progress, many countries continue to experience high burdens of trachoma, including Ethiopia, where 61 million people live in trachoma-endemic districts, accounting for 59% of the global at-risk population.^1^

To achieve the elimination of trachoma as a public health problem, as targeted by the global road map for neglected tropical diseases 2021–2030,^2^ the WHO recommends the implementation of the surgery, antibiotics, facial cleanliness, and environmental improvement (SAFE) strategy, which includes community-wide distribution of azithromycin, also known as mass drug administration (MDA), in areas with a prevalence of trachomatous inflammation—follicular (TF) ≥5% among children aged 1 to 9 years.^3^ Mass drug administration is typically conducted annually; however, evidence from modeling and cross-sectional studies suggests that annual MDA alone may be insufficient to achieve elimination as a public health problem in some settings, such as districts with high prevalence (>40% TF) at baseline or areas with persistent trachoma, such as Amhara, Ethiopia.^4^^–^^6^ Persistent trachoma is defined as any district that remains above the 5% elimination threshold for TF following MDA intervention and at least two trachoma impact surveys.^7^

To address the challenge of persistent trachoma, modified MDA strategies have been proposed, including more frequent than annual (MFTA) MDA.^7^ In Ethiopia, the Ministry of Health proposed implementing an MFTA MDA strategy that provided one standard treatment of community-wide MDA to all eligible individuals, followed by an additional MDA treatment targeting children aged 6 months to 9 years, 4 to 6 weeks later. The “child MDA” (CMDA) strategy, as it was designated in Ethiopia, intended to enhance the impact of MDA by targeting those who harbor the greatest levels of infection.^8^^,^^9^

**Figure 1.**
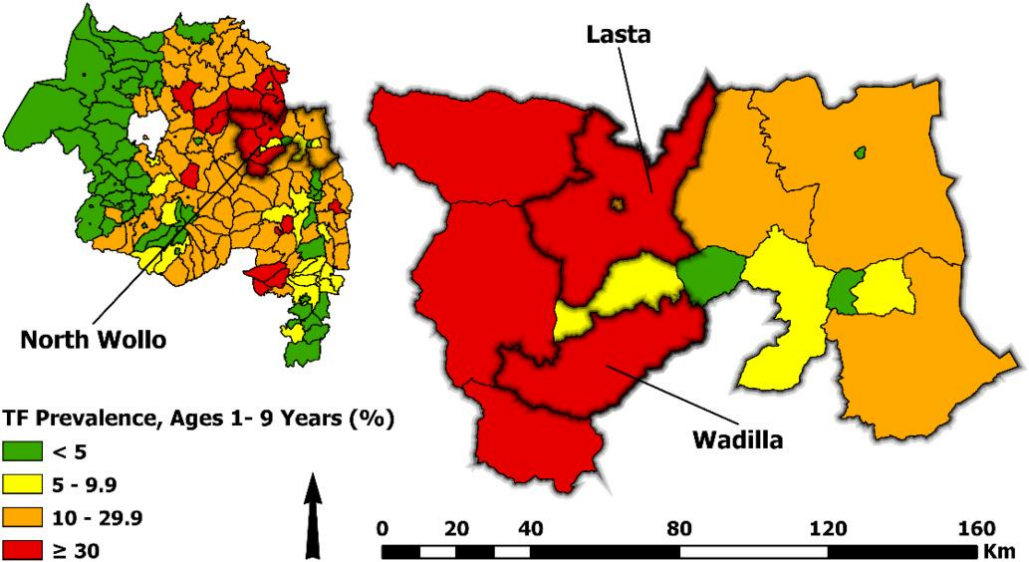
Map of the Amhara Region, Ethiopia, highlighting the districts included as part of the child mass drug administration strategy in 2023.

In 2023, the Amhara Trachoma Control Program implemented this CMDA strategy in two districts, contributing to the Ministry of Health’s wider six-district pilot for MFTA MDA across the country. As part of the CMDA strategy implementation in Amhara, the Trachoma Control Program collected comprehensive cost data. The aim for the present study was to determine the cost of the CMDA strategy implementation in two districts, Lasta and Wadilla (Figure 1), to support decision-making for further scale-up throughout Ethiopia and inform future analyses on the cost-effectiveness of this strategy.

## MATERIALS AND METHODS

### Child MDA strategy methods.

In May 2023, the Amhara Trachoma Control Program implemented the CMDA strategy in Lasta and Wadilla districts. The initial community-wide MDA treatment was implemented according to national guidelines, which align with recommendations from the International Trachoma Initiative Zithromax® Management Guide.^10^ The guide recommends that all individuals aged 15 years or older be offered a full adult dose of 4 azithromycin tablets; all individuals taller than 120 cm and between 7 and 15 years of age be offered 3 or 4 azithromycin tablets (determined by height); all children from 6 months through 6 years of age, as well as any child with difficulties or discomfort swallowing tablets, receive powder for oral suspension (POS; dosage according to height); and children under 6 months of age be provided tetracycline eye ointment (TEO). During the subsequent child-only MDA treatment targeting children aged 6 months through 9 years, the standard treatment guidelines were also followed, although no TEO was distributed, as children under 6 months of age were ineligible for the additional treatment.

Preparation for MDA implementation began 1 month before the commencement of MDA activities and included support from employees of The Carter Center, who coordinated with relevant district and zonal health department offices; the estimated number of days spent preparing for MDA implementation by job title is available in Supplemental Table 1.

From April 25 to April 26, 2023, training was provided to district and zonal-level coordinators, who were responsible for cascading the training to others within the districts involved in MDA implementation. During the district-level training held from May 12 to May 13, 2023, coordinators trained field supervisors, team leaders, and community leaders. In total, 42 people were trained at the zonal level in North Wollo; 104 people were subsequently trained by the coordinators in Lasta; and 92 people were trained in Wadilla. All trainers and trainees received per diem payments for participating in the training and for their travel time to the training sites; they also received lunch and refreshments during the training. Further details about training costs are available in Supplemental Table 2.

To sensitize the community and raise awareness about the upcoming MDA activities, a zonal advocacy workshop was held on April 24, 2023. Political leaders from both the zonal and district levels attended the workshops. Other community sensitization efforts included engaging local schools and disseminating radio messages, which were broadcast during the community-wide MDA treatment in each district. Community leaders also informed their respective communities about the MDA implementation and advocated for community participation. Additionally, a half-day orientation was conducted for supervisors and coordinators before the child-only MDA treatment. Drugs for both community-wide and child-only distributions were transported from the central store to each district simultaneously by the Ethiopian Pharmaceutical Supply Service (formerly the Ethiopian Pharmaceutical Supply Agency).

Mass drug administration was implemented in the community by distribution teams that consisted of one health extension worker (HEW), who is a paid government healthcare worker, acting as a team leader, and one to three volunteers from the Health Development Army (HDA), a network of community volunteers working together to prevent disease and improve health through community empowerment and participation.^11^ Distribution teams were responsible for disseminating health education messages, measuring the height of MDA participants, dispensing drugs, and recording treatment data. Supervisors, selected by district health offices, ensured that MDA activities were conducted according to protocol, provided additional training to distribution teams when gaps were observed, and submitted performance reports to district coordinators.

### Lasta district.

In Lasta, 77 distribution teams were deployed to implement the CMDA strategy, beginning with the initial community-wide MDA treatment that took place from May 18 to May 24, 2023 (7 days). A total of 128,939 individuals were treated in Lasta, out of a target population of 147,270; of those treated, 95,828 received azithromycin tablets, 30,749 received azithromycin POS, and 2,362 received TEO. Overall, 88% of the targeted population was treated (Table 1).

**Table 1:** Summary of people treated and coverage rates for each mass drug administration treatment during implementation of the child mass drug administration strategy in Lasta and Wadilla, Ethiopia, 2023

District	Target Population	People Treated	Population Coverage
Lasta community-wide MDA treatment	147,270	128,939	88%
Lasta child-only MDA treatment (6 months to 9 years)	42,856	39,236	92%
Wadilla community-wide MDA treatment	137,681	129,892	94%
Wadilla child-only MDA treatment (6 months to 9 years)	40,065	39,356	98%

MDA = mass drug administration.

For the subsequent child-only treatment, 77 distribution teams were deployed from June 17 to June 21, 2023 (5 days). A total of 39,236 children were treated, out of a target population of 42,856; of those, 1,720 received azithromycin tablets, and 37,516 received azithromycin POS. Overall, 92% of the targeted population was treated (Table 1).

### Wadilla district.

In Wadilla, 66 distribution teams were deployed to implement the CMDA strategy, beginning with the initial community-wide MDA treatment that took place from May 19 to May 25, 2023 (7 days). A total of 129,892 individuals were treated in Wadilla, out of a target population of 137,681; of those treated, 98,464 received azithromycin tablets, 28,794 received azithromycin POS, and 2,634 received TEO. Overall, 94% of the targeted population was treated (Table 1).

For the subsequent child-only treatment, the 66 distribution teams were deployed from June 18 to June 22, 2023 (5 days). A total of 39,356 children were treated, out of a target population of 40,065; of those treated, 7,573 received azithromycin tablets, and 31,783 received azithromycin POS. Overall, 98% of the targeted population was treated (Table 1).

A review meeting was held in each district after completion of each treatment (community-wide and child-only). The review meeting participants consisted of all personnel trained during the zonal- or district-level trainings to support MDA implementation. These included employees of The Carter Center, health department staff, administrative officers from the district health office, HEWs, supervisors, and community leaders.

### Costing methods.

The present study was designed using the Global Health Cost Consortium Reference Case and Global Health Cost Consortium Principles and Methods Checklist.^12^^,^^13^

A bottom-up micro-costing analysis was conducted to document the costs and quantities of all elements required for both MDA treatments of the CMDA strategy implemented in Lasta and Wadilla. Real-world net implementation costs were entered into a costing database developed in Microsoft Excel (Microsoft Corp., Redmond, WA) using the payer perspective. The district served as the unit of analysis in the present study.

The primary outcomes of the current study were the total cost, cost per person treated, and cost per treatment of the CMDA strategy in Lasta and Wadilla. The combined cost was calcualted for the overall implementation of the CMDA strategy in both Lasta and Wadilla, as well as the costs per district. Additionally, the cost of community-wide MDA treatment and the additional costs associated with the second treatment of the CMDA strategy solely targeting children aged 6 months to 9 years were estimated.

The cost per person treated was calculated by dividing the total cost by the total number of individuals treated in the community-wide MDA treatment. The cost per treatment was calculated by dividing the total cost by the total number of treatments distributed overall.

To calculate the cost of community-wide MDA treatment alone, costs associated with child-only treatment distribution were removed. Specifically, the time spent planning MDA activities was reduced by 2 days per staff member, on the basis of programmatic information elicited from interviewed experts. Costs specifically associated with additional orientation, community sensitization, implementation, supervision, and review meetings related only to the child-only MDA treatment were also removed. Drug transportation costs were not adjusted because the child-only MDA treatment did not increase those costs. The cost of the child-only MDA treatment was calculated by subtracting the cost of the community-wide MDA treatment from the total cost of all MDA activities. The additional cost per child treated in the child-only MDA was calculated by dividing the additional cost of the child-only MDA treatment by the number of children treated in the second round.

Societal costs related to the intervention, such as productivity losses associated with attending MDAs, were not included. Costs incurred by other stakeholders, such as Ministry of Health officials, were also excluded.

Both the financial and economic costs were calculated for all primary outcomes. The financial cost analysis included all elements that incurred a monetary expenditure and did not account for elements such as donated medicines or volunteer labor. In contrast, the economic cost analysis included both the cost of elements that incurred a monetary expenditure, as well as the value of other resources, such as donated medicines and volunteer labor. Consequently, the financial cost can be interpreted as the financial expenditure required to implement MDA, whereas the economic cost can be interpreted as the value of resources required to implement MDA.^14^

To calculate the financial costs of implementing the CMDA strategy, the cost and quantity of all resources paid for by The Carter Center were documented. The cost of capital items, such as laptops, was estimated using straight-line depreciation, whereby the total cost of the item was divided by the number of years it was deemed useful.^12^ The financial cost does not include the cost of azithromycin tablets or POS, which are donated by Pfizer Inc. (New York, NY) through the International Trachoma Initiative. The financial cost estimation includes the cost of TEO, which is purchased by The Carter Center. The average quantity of azithromycin tablets, azithromycin POS, and TEO in a single dose is presented in Supplemental Table 3.

To calculate the economic cost of implementing the CMDA strategy, the costs of all supplies purchased by The Carter Center, as well as the cost of donated medicines and labor from HDA volunteers, were considered.^15^ The value of donated medicines was estimated to be $4.55 per azithromycin tablet and $0.58 per ml of POS, after adjusting for the Task Force for Global Health’s 2024 fair market value of $4.66 per azithromycin tablet and $0.59 per ml of POS.^16^^,^^17^ The cost of HDA volunteers was estimated at $5.19 per day for each volunteer, consistent with per diem payments for team leaders. The economic cost of capital elements were annualized over their estimated useful life (estimated by the Carter Center staff), and a 3% discount rate was applied using the annualization factor.^18^ All costs are presented in 2023 US dollars.^16^ An exchange rate of US$1 = 57.21 Ethiopian Birr was applied.^19^

### Cost breakdown.

The cost categories and elements included in the analysis are presented in Table 2. The total cost of CMDA strategy implementation was calculated using the price and quantity of all listed elements, which were identified retrospectively through a review of the Carter Center’s financial records and consultation with Carter Center staff. As in previous studies, an additional 25% was added to the total financial cost to account for central program and overhead costs.^20^^,^^21^

**Table 2:** Cost categories and elements in the child mass drug administration strategy implementation in Lasta and Wadilla, Ethiopia, 2023

Category	Central Program	Training	Community Sensitization	Drug Transportation	Drug Distribution	Review Meetings	Supervision
Office costs	X	–	–	–	–	–	–
Computer equipment	X	–	–	–	–	–	–
Accommodation and/or venue hire	–	X	–	–	–	X	X
Salaried labor	X	X	X	–	X	X	X
Per diems	–	X	X	–	X	X	X
Azithromycin	–	–	–	–	X	–	–
Tetracycline eye ointment	–	–	–	–	X	–	–
Fuel	–	X	–	X	X	X	X
Vehicles	–	X	–	X	X	X	X
Lunch and/or refreshments	–	X	X	–	–	X	–

Central program costs, such as laptops, were considered central costs that benefited all trachoma activities coordinated by the Trachoma Control Program. As such, only a proportion of capital item elements under central program costs were attributed to CMDA-related activities in Lasta and Wadilla. Program staff estimated that 15% of capital item costs were allocated to CMDA-related activities in 2023. Staff salaries were converted into day rates and applied to each cost category on the basis of the number of days an employee supported CMDA-related activities.

When unit costs and quantities were unavailable, gross costs were applied, or a top-down approach was used to divide the total cost of expenditure, and the cost was allocated across relevant categories. Most notably, the cost of fuel was allocated using the proportion of days of vehicle use for each category. Other gross costs included training materials, such as flipcharts and stationery, as well as lunch and refreshment packages. Further information is available in Supplemental Table 2.

### Sensitivity analyses.

Univariate sensitivity analyses were used to explore how programmatic changes affected the financial costs of implementing the CMDA strategy.^22^ The following key parameters were evaluated: 1) increasing the costs of implementation and supervision by 50% to assess the financial impact of extending the length of MDA implementation as a result of unforeseen programmatic delays or efforts to improve coverage; 2) estimating the impact of varying coverage levels by presenting the overall cost per person in the two districts at 80%, 90%, and 100% coverage; 3) reducing central program costs from 25% to 15% of the total financial costs; 4) removing the cost of the first review meeting after initial community-wide MDA treatment in Lasta and Wadilla, which may not be required in the future when the CMDA strategy is more established; and 5) removing central program costs, including salaries, to identify direct implementation expenses.

## RESULTS

### Financial cost analysis.

The total financial cost of implementing the CMDA strategy in Lasta and Wadilla was $106,427, corresponding to an overall financial cost per person treated of $0.41 ($0.48 in Lasta and $0.34 in Wadilla; Table 3). In total, 337,423 treatments were distributed across the two districts and both MDA treatments (168,175 in Lasta and 169,248 in Wadilla), equating to an overall financial cost per treatment of $0.32 ($0.37 in Lasta and $0.26 in Wadilla). A breakdown of costs by category is available in Supplemental Table 4.

**Table 3:** Financial cost results for the child mass drug administration strategy implementation, Lasta and Wadilla, Ethiopia, 2023

Financial Cost	Lasta and Wadilla	Lasta	Wadilla
Total financial cost of CMDA strategy implementation	$106,427	$61,978	$44,449
Cost of CMDA strategy per person treated	$0.41	$0.48	$0.34
Cost of CMDA strategy per treatment	$0.32	$0.37	$0.26
Cost of community-wide MDA treatment	$71,147	$41,737	$29,410
Cost per person of community-wide MDA treatment	$0.27	$0.32	$0.23
Additional cost of child-only MDA treatment	$35,280	$20,241	$15,039
Additional cost per child of child-only MDA treatment	$0.45	$0.52	$0.38

CMDA = child mass drug administration; MDA = mass drug administration.

When excluding the child-only MDA treatment, the financial cost for the initial community-wide MDA treatment in both districts was $71,147, equating to an overall cost per person treated of $0.27 ($0.32 in Lasta and $0.23 in Wadilla). The additive cost for the subsequent child-only treatment, which treated 78,592 children aged 6 months to 9 years across both districts, was $35,280, or an additional cost of $0.45 per child treated. If broken down by district, the additional financial costs of the child-only treatment, which served 39,236 children in Lasta and 39,356 children in Wadilla, were $0.52 and $0.38, respectively.

### Economic cost analysis.

The total economic cost to implement the CMDA strategy in Lasta and Wadilla, which includes the full value of resources such as donated medicines and volunteer labor, was $4,357,209 ($2,147,596 in Lasta and $2,209,613 in Wadilla), at an economic cost of $16.83 per person treated ($16.66 in Lasta and $17.01 in Wadilla; Table 4). The overall economic cost per treatment of the CMDA strategy was $12.91 ($12.77 in Lasta and $13.06 in Wadilla). When removing the costs associated with child-only MDA treatment, the economic cost of standard community-wide MDA was estimated at $3,795,087 ($1,887,571 in Lasta and $1,907,516 in Wadilla), equating to a cost per person of $14.66 ($14.64 in Lasta and $14.69 in Wadilla). The additive economic cost for subsequent child-only MDA treatment to 78,592 children aged 6 months to 9 years across both districts was $562,122 ($260,025 in Lasta and $302,098 in Wadilla), or an additional $7.15 per child treated ($6.63 in Lasta and $7.68 in Wadilla).

**Table 4:** Economic cost results for the child mass drug administration strategy implementation, Lasta and Wadilla, Ethiopia, 2023

Economic Cost	Lasta and Wadilla	Lasta	Wadilla
Total financial cost of CMDA strategy implementation	$4,357,209	$2,147,596	$2,209,613
Cost of CMDA strategy per person treated	$16.83	$16.66	$17.01
Cost of CMDA strategy per treatment	$12.91	$12.77	$13.06

CMDA = child mass drug administration.

### Sensitivity analysis.

When the number of days to implement the CMDA strategy was doubled, the total financial cost of MDA implementation across both districts increased from $106,427 to $130,841 (a 23% increase). The financial cost per person treated increased from $0.41 to $0.51.

The total cost per person treated and the total cost per treatment after adjusting the coverage rate while keeping the number of treatment days constant is shown in Table 5. The cost per person treated range could change by up to $0.11 per person, depending on the MDA coverage rate.

**Table 5:** Financial cost per person treated at various coverage rates for the child mass drug administration strategy implementation in Lasta and Wadilla, Ethiopia, 2023

Coverage Rate	Combined Financial Cost Per Person Treated	Lasta Cost Per Person Treated	Wadilla Cost Per Person Treated
80%	$0.47	$0.53	$0.40
90%	$0.41	$0.47	$0.36
100%	$0.37	$0.42	$0.32

When the percentage of central program costs was reduced from 25% to 15%, the total financial cost decreased from $106,427 to $97,913 (8%). The total cost per person treated decreased from $0.41 to $0.38, and the total cost per treatment decreased from $0.32 to $0.29. When we removed the review meetings conducted after the community-wide MDA, the total financial cost decreased from $106,427 to $94,895 (11%), the cost per person treated decreased from $0.41 to $0.37, and the total cost per treatment decreased from $0.32 to $0.28. When only implementation costs were considered and central program costs and salaries were removed, the total cost for implementation decreased from $106,427 to $77,794. The cost per person treated decreased from $0.41 to $0.30, and the cost per treatment decreased from $0.32 to $0.23 (a breakdown of the cost per cost category is available in Supplemental Table 5).

## DISCUSSION

As part of implementing the CMDA strategy, the total cost, cost per person treated, and cost per treatment of both the initial community-wide treatment and the subsequent child-only treatment targeting children aged 6 months to 9 years in the Lasta and Wadilla districts in Amhara, Ethiopia, were presented. The study analysis revealed that the financial cost per person for implementing the CMDA strategy in Lasta and Wadilla was $0.27 for the initial community-wide treatment and an additional financial cost of $0.45 per child for the subsequent child-only treatment. This cost data will be useful to policymakers and implementers in Ethiopia as they consider expanding the CMDA strategy to accelerate progress toward its 2030 elimination target.

The cost per person treated in the child-only MDA treatment is notably higher than that of the initial community-wide treatment. This is, in part, due to economies of scale and a larger number of people treated in the community-wide distribution compared with the child-only treatment within the same geographic areas.^23^ However, in addition to the cost efficiencies already realized through the CMDA strategy implementation, such as minimal additional training days and the ability to transport drugs for both MDA treatments (community-wide and child-only) in a single trip, further efficiencies are likely to be achieved as the program matures. This may include a reduced number of implementation days as distribution teams and communities become more familiar with the intervention; review meetings could also be consolidated after both treatments have been distributed, which could reduce the total costs by ∼12%.

Additionally, although the cost per child treated in child-only MDA is higher than the cost per person treated in community-wide MDA, treating children, who are known to harbor the highest trachoma burden, may have a greater overall impact on addressing the trachoma burden. The increased cost to treat this population may result in long-term cost savings if districts achieve elimination thresholds more quickly than with standard community-wide MDA. Therefore, the higher per capita cost associated with child-only MDA treatment should be evaluated not only in financial terms but also in terms of its potential epidemiological impact. Further research is warranted to assess the overall cost-effectiveness of this targeted approach, including its long-term benefits and potential to accelerate progress toward trachoma elimination goals.

Primary outcomes were calculated separately for each district to highlight cost variation across settings. Notably, the present study revealed that the cost per person to implement the CMDA strategy in Lasta was $0.48, whereas the cost per person in Wadilla was $0.34. This was driven by several factors, including an additional $5,661 spent on review meetings in Lasta, $3,695 spent on supervision, and $3,779 spent on training. The increased costs of training, supervision, and review meetings were largely due to the greater number of distribution teams required and the longer travel time for distribution teams in Lasta, which, in turn, increased the cost of per diem payments. Program staff report that this is largely attributable to the difficult terrain in Lasta compared with Wadilla, which slows implementation. Furthermore, the geographical size of Lasta, which is 1,119 km^2^ compared with 855.3 km^2^ in Wadilla,^24^^,^^25^ could also impact implementation because of its lower population density and greater distances required to reach similar target populations.

The current study revealed that the average financial cost per person treated as part of the CMDA strategy in Lasta and Wadilla combined was $0.41. In this analysis, the number of people treated in both districts was used as the denominator; therefore, the unit cost of $0.41 can be compared with findings from other studies that include the unit cost for community-wide MDA. Importantly, the present study revealed that although the cost per person to implement the CMDA strategy was higher than that of the standard community-wide MDA in Amhara, the cost of implementing the CMDA strategy in Lasta and Wadilla was still less expensive than a single treatment of annual community-wide MDA in several other settings where costing analyses have been conducted.^20^^,^^26^^,^^27^ For example, in neighboring South Sudan, the cost per person treated as part of community-wide MDA ranged from $1.08 to $1.22.^28^ This highlights the variability of costs across geographical settings and program structures, such as the use of volunteers or paid drug distributors, as well as the potential for increased cost-effectiveness of MDA for trachoma in Amhara, Ethiopia.

The sensitivity analysis also revealed the impact of economies of scale on the cost per person treated, with the cost per person decreasing as treatment coverage increased when all costs are held constant. Although achieving high coverage often incurs additional expenditure, programs should expect variation in coverage from year to year, which will affect the cost per person treated. As such, it is useful to consider a range of implementation costs when budgeting, rather than a specific cost from a singular context. The sensitivity analysis further revealed that doubling the implementation time for CMDA increased the total costs by 14–17%. Therefore, it might be cost-effective for a program to extend CMDA to reach a greater number of people, which could result in a greater reduction in trachoma prevalence. Additionally, the sensitivity analysis revealed that the implementation cost for the CMDA strategy, after accounting for central program costs and salaries, is ∼$0.30 per person treated, or $0.23 per treatment. This information is useful for program managers developing budgets that do not need to account for staff salaries or central program infrastructure.

The present study had several limitations. Notably, it was limited by uncertainty in the population denominator, which is used to define the target population and assess MDA coverage; this is a challenge often encountered by trachoma programs.^29^ The current study did not include costs associated with technical support from The Carter Center headquarters in Atlanta, which was determined to be minimal and therefore included in the 25% central program costs. Lastly, the results of the present study are specific to Lasta and Wadilla. Generalizing these results to other settings outside the Amhara region of Ethiopia should be performed cautiously, taking into consideration labor costs, geographical and environmental factors, population demographics, population density, and other factors that could influence programming costs. Furthermore, in the study analysis, the second treatment occurred ∼1 month after the first, in line with the CMDA strategy established by the Ministry of Health. Other countries may opt for different enhanced treatment approaches, including longer time intervals between treatments, which may entail cost implications related to longer drug storage times, or the need to plan for multiple drug transports or conduct refresher training.

The results, however, provide a useful indication of the range of costs that might be incurred in similar contexts and offer valuable information to inform further research on MFTA MDA. If MFTA strategies are feasible and more effective at reducing transmission than a single annual community-wide MDA treatment, then the increased short-term programmatic costs could yield long-term savings alongside improved health outcomes.

Lastly, this costing analysis was used to assess the economic costs—including estimating the monetary value of donated medicines and volunteer time—of implementing the CMDA strategy in two districts in Amhara, Ethiopia. Assessing these costs is important, as scaling up CMDA to other areas will increase the need for additional HDA members to support HEWs with MDA activities; the economic analysis helps quantify the opportunity cost of those resources. In addition, assigning value to donated medicines helps quantify the additional resources national trachoma programs receive beyond programmatic investments. This reinforces the case for continued investment and provides a compelling advocacy message about the leverage gained through the drug donation program. At the same time, it underscores the importance of accelerating trachoma elimination during the drug donation program period so that progress is not threatened.

To estimate the economic value of Pfizer-donated azithromycin, the fair market price calculated by the Task Force for Global Health was used. The analysis highlights the substantial value of donated medicines to the global trachoma program, with more than $4.2 million worth of azithromycin leveraged through the CMDA strategy implementation in Lasta and Wadilla alone. However, the literature remains inconsistent on the data sources used to estimate the economic value of azithromycin, making cost comparisons across studies challenging. The authors of one study referenced the International Medical Products Price Guide,^30^ those of another relied on online market prices,^27^ and those of a third study excluded the cost of the drug altogether.^20^ This can have a significant impact on the estimated economic costs of MDA. Moving forward, adopting a standardized methodology for costing studies related to trachoma would facilitate more accurate comparisons, support robust economic evaluations, and enhance advocacy messaging by clearly demonstrating a consistent economic value of donated medicines.^28^

## CONCLUSION

The present study revealed that the cost of CMDA strategy implementation ranged from $0.34 to $0.48 per person treated across the two districts. The results of the study will be useful when incorporated with effectiveness data to inform subsequent budget impact or cost-effectiveness analyses and support decision-making about MFTA MDA in Ethiopia.

## Supplemental Materials

10.4269/ajtmh.25-0498 Supplemental Materials
