# Supplementary material for: The Cost of Annual and More Frequent Than Annual Mass Drug Administration for Trachoma in Two Districts in Amhara, Ethiopia
Source: Am J Trop Med Hyg. 2026 Jan 20;114(3):445–52. doi: 10.4269/ajtmh.25-0498 (PMC12964873; doi:10.4269/ajtmh.25-0498)
Supplement: Supplemental Materials [file tpmd250498.SD1.pdf]

Supplementary material

Supplemental Table S1: Estimated number of days spent preparing for MDA implementation.

Job title	Estimated Number of Days Spent Preparing for MDA Implementation
Project Coordinator	7
Project Coordinator	7
Program Officer	5
Sub-Regional Manager	5

Supplemental Table S2: Overview of financial cost of ingredients included in the costing analysis of the CMDA strategy implementation in Lasta and Wadilla, Ethiopia, 2023.

Cost Ingredients by Cost Category	Total Cost
**CENTRAL PROGRAM**	
Staff Salary Costs	$1,336.69
Laptops	$130.76
Airtime Package	$63.13
**TRAINING**	
**Zonal Training**	
Staff Salary Costs	$1,464.55
Per Diems	$3,500.65
Venue and Refreshments	$286.85
Vehicles and Fuel	$848.04
**Lasta Training**	
Staff Salary Costs	$258.22
Per Diems	$6,165.57
Stationary	$57.08
Venue and Refreshments	$1,315.42
Vehicles and Fuel	$810.63
**Lasta Child-Only MDA Orientation**	
Per Diems	$707.03
Venue and Refreshments	$126.38
**Wadilla Training**	
Staff Salary Costs	$134.83
Per Diems	$3,433.70
Stationary	$50.20
Venue and Refreshments	$394.27
Vehicles and Fuel	$815.08
**Wadilla Child-Only MDA Orientation**	
Per Diems	$436.04
Venue and Refreshments	$118.15
**COMMUNITY SENSITIZATION**	
**Zonal Advocacy**	
Staff Costs	$2,016.59
**Lasta Community Sensitization**	
General MDA Commodities	$369.34
Child-Only MDA Commodities	$894.20
**Wadilla Community Sensitization**	
General MDA Commodities	$369.04
Child-Only MDA Commodities	$586.95
**DRUG TRANSPORTATION**	
Lasta	$185.19
Wadilla	$179.06
**DRUG DISTRIBUTION**	

**Lasta Community-Wide CMDA Drug Distribution**	
Supervision-Staff Salary Costs	$1,451.77
Supervision-Per Diems	$3,474.69
Distribution-Per Diems	$4,103.35
Azithromycin (economic cost)	$53,493.11
POS (economic cost)	$11,284.88
TEO	$1,086.52
Supervision-Vehicles and Fuel	$3,450.62
**Lasta Child-Only MDA Drug Distribution**	
Supervision-Staff Salary Costs	$866.24
Supervision-Per Diems	$2,378.58
Distribution-Staff Per Diems	$2,687.73
Azithromycin (economic cost)	$758.00
POS (economic cost)	$16,522.05
Supervision-Vehicles and Fuel	$2,026.58
**Wadilla Community-Wide MDA Drug Distribution**	
Supervision-Staff Salary Costs	$297.53
Supervision-Per Diems	$2,051.32
Distribution-Per Diems	$3,523.96
Azithromycin (economic cost)	$ 54,964.57
POS (economic cost)	$10,567.40
TEO	$1,211.64
Supervision-Vehicles and Fuel	$3,332.55
**Wadilla Child-Only MDA Drug Distribution**	
Supervision-Staff Salary Costs	$435.31
Supervision-Per Diems	$1,456.28
Distribution-Per Diems	$1,211.64
Azithromycin (economic cost)	$3,337.42
POS (economic cost)	$13,997.23
Supervision-Vehicles and Fuel	$2,380.39
**REVIEW MEETINGS**	
**Lasta Community-Wide MDA Review Meeting**	
Per Diems	$5,174.11
Utilities	$901.90
**Lasta Child-Only MDA Review Meeting**	
Per Diems	$5,325.04
Utilities	$942.58
**Wadilla Community-Wide MDA Review Meeting**	
Per Diems	$2,748.84
Utilities	$400.95
**Wadilla Child-Only MDA Review Meeting**	
Per Diems	$2,907.72
Utilities	$625.13

MDA = mass drug administration; POS = Powder Oral Suspension; TEO = tetracycline eye ointment

Supplemental Table S3: Average quantity of azithromycin, azithromycin POS, and TEO in a single dose administered during the CMDA strategy implementation in Lasta and Wadilla, Ethiopia, 2023.

CMDA Strategy Treatment Events	Azithromycin (tablets)	Azithromycin POS (ml)	TEO (tubes)
Community-Wide MDA Treatment	3.8	10	2
Child-Only MDA Treatment	3.0	10	-

Supplemental Table S4: Total financial and economic cost per cost category and the percentage of each category against total costs of the CMDA strategy implementation in Lasta and Wadilla, Ethiopia, 2023.

Cost Category	Total Financial Cost in Lasta and Wadilla	Percentage of Cost	Total Economic Cost in Lasta and Wadilla	Percentage of Cost
Central Program	$22,816	21%	$22,279	8%
Training	$19,535	18%	$19,535	7%
Community Sensitization	$5,624	5%	$5,624	2%
Drug Transportation	$364	0%	$364	0%
Drug Distribution	$15,460	15%	$192,628	68%
Review Meetings	$19,026	18%	$19,026	7%
Supervision	$23,602	22%	$23,602	8%

Supplemental Table S5: Total financial cost per cost category when central program costs and salaries were excluded in the CMDA strategy implementation in Lasta and Wadilla, Ethiopia, 2023.

**Cost Category**	**Total Financial Cost in Lasta and Wadilla**	**Percentage of Cost**
Central Program	$0	0%
Training	$17,677	23%
Community Sensitization	$5,464	7%
Drug Transportation	$364	0%
Drug Distribution	$15,308	20%
Review Meetings	$18,430	24%
Supervision	$20,551	26%
